# Association between triglyceride glucose-body mass index and diabetic kidney disease in hospitalized patients with type 2 diabetes mellitus: a retrospective cross-sectional study

**DOI:** 10.3389/fmed.2026.1840269

**Published:** 2026-09-24

**Authors:** Sixu Xin, Xiaomei Zhang, Xin Zhao, Jianbin Sun

**Affiliations:** Department of Endocrinology, Peking University International Hospital, Beijing, China

**Keywords:** diabetes mellitus, diabetic kidney disease, triglyceride glucose-body mass index, TyG-BMI index, type 2

## Abstract

**Objectives:**

To analyze the association between the triglyceride glucose-body mass index (TyG-BMI) and diabetic kidney disease (DKD) in patients with type 2 Diabetes Mellitus (T2DM).

**Methods:**

A single-center retrospective cross-sectional study was conducted enrolling 1,966 patients diagnosed with T2DM. General clinical data, as well as data on medication use, smoking history, alcohol consumption, and comorbidities, were collected. According to DKD diagnostic criteria, participants were divided into the non-DKD group (*n* = 1,359) and the DKD group (*n* = 607). Subgroup analyses were performed according to DKD clinical stages. Multivariate logistic regression analysis was employed to investigate the association between the TyG-BMI index and DKD in T2DM patients, and receiver operating characteristic (ROC) curve analysis was used to evaluate the discriminatory ability of TyG-BMI for DKD.

**Results:**

Compared to the Non-DKD group, patients in the DKD group exhibited significantly higher age, DM duration, SBP, DBP, WC, WHR, BMI, sCr, UACR, FPG, 2hPPG, HbA1c, FCP, TG, and the TyG-BMI index (*P* < 0.05). Conversely, HDL-C, e-GFR, and ALT were significantly lower in the DKD group (*P* < 0.05). Subgroup analysis revealed that TyG-BMI index showed a gradual increasing trend with increasing DKD severity, but the difference between the two subgroups was not statistically significant (*P* > 0.05). Multivariate logistic regression analysis, after adjusting for confounders including age, sex, diabetes duration, SBP, comorbidities, and medication history, identified the TyG-BMI index as an independent factor associated with DKD, with each 10-unit increase in TyG-BMI associated with an 8.5% increase in DKD risk (OR = 1.085, 95% CI: 1.059–1.112, *P* = 0.000). When TyG-BMI index was stratified by quartiles, logistic regression analysis demonstrated that the risk of DKD gradually increased with higher TyG-BMI levels, and the trend test indicated a significant dose–response relationship (*P* for trend < 0.001). ROC curve analysis showed that the combined model (including TyG-BMI, demographic and clinical variables) achieved an area under the curve (AUC) of 0.709 (95% CI: 0.683–0.734) for identifying DKD, with an optimal predicted probability cut-off of 0.330 (corresponding to a raw TyG-BMI index value of 315.962), a sensitivity of 58.4%, and a specificity of 73.4%. The AUC for TyG-BMI alone was 0.574 (95% CI: 0.547–0.601), and for BMI alone was 0.552 (95% CI: 0.524–0.580). The DeLong test demonstrated that the AUC of the combined model was significantly superior to that of TyG-BMI alone and BMI alone (both *P* < 0.001), indicating that the addition of other clinical variables significantly improved the discriminative ability of the model.

**Conclusion:**

This study identified a significant association between the TyG-BMI index and DKD in patients with T2DM, with a dose-response trend. As a simple composite index derived from TG, FBG, and BMI, TyG-BMI index may serve as an auxiliary reference indicator for DKD risk assessment in T2DM patients, facilitating the identification of high-risk individuals. However, due to the selection bias inherent in the hospitalized population and the cross-sectional design, these findings require further validation in community-based cohorts.

## 1 Introduction

With the improvement of economic standards, changes in lifestyle, and the aging of the population, the global incidence of diabetes mellitus (DM) has been increasing. According to the International Diabetes Federation (IDF) diabetes Atlas (1), the global diabetes prevalence among individuals aged 20–79 years was estimated at 10.5% (536.6 million people) in 2021 and is projected to increase to 12.2% (783.2 million people) by 2045. China has the largest number of individuals with diabetes worldwide. According to the American Diabetes Association (ADA) 2010 diagnostic criteria, the prevalence of DM in China increased from 10.9% in 2013 to 12.4% between 2018 and 2019 (2), affecting more than 140 million individuals (3). Diabetic kidney disease (DKD), as one of the major chronic microvascular complications, occurs in 20%–40% of patients with DM (4), and represents the leading cause of end-stage renal disease (ESRD) (5). In addition, among patients with either type 1 or type 2 diabetes, the presence of chronic kidney disease (CKD) markedly increases cardiovascular risk and healthcare costs (6). Currently, no effective pharmacological therapies are available to reverse DKD, and once its pathological process initiates, it is difficult to halt its progression. Therefore, early detection of renal injury in diabetic patients and timely implementation of appropriate interventions are critically important (7). Insulin resistance (IR) is one of the primary pathophysiological mechanisms underlying type 2 diabetes mellitus (T2DM) and has been demonstrated to induce glucose metabolism disorder, inflammation and oxidative stress responses, thereby increasing the risk of both microvascular and macrovascular complications in DM. IR and DKD are mutually causative and interwoven throughout the entire course of DKD. They exacerbate each other as DKD progresses, accelerating its advancement toward ESRD (8). Consequently, the prediction of IR may hold significant value for the early detection of diabetic kidney injury.

The hyperinsulinemic-euglycemic clamp (HEGC), considered the gold standard for measuring IR, is difficult to apply in large population studies and clinical settings due to its high cost, time consuming nature, and complexity. To address this limitation, the homeostatic model assessment of insulin resistance (HOMA-IR) was developed to evaluate β-cell function and IR. However, HOMA-IR requires measurement of fasting insulin levels, making it less suitable for general population screening. Given these limitations, the use of biomarkers sensitive to IRas clinical tools for assessing metabolic health may offer practical advantages in both research and clinical practice. In recent years, the triglyceride-glucose (TyG) index has garnered widespread attention as a simple surrogate marker for evaluating insulin resistance. The TyG index is derived from triglyceride (TG) and fasting plasma glucose (FPG) levels and demonstrates good correlation with both HEGC and HOMA-IR (9). Multiple studies have confirmed that the TyG index is independently associated with DKD risk (10–12) and can serve as a reliable predictor of CKD risk (13). However, the TyG index solely reflects glucolipid metabolic disorders and does not incorporate anthropometric indicators that reflect the degree of obesity. Given that obesity is a significant risk factor for both IR and DKD, TyG-derived indices that integrate anthropometric measurements may possess superior predictive value. The triglyceride glucose-body mass index (TyG-BMI), a composite parameter derived from FPG, TG, and body mass index (BMI), has been demonstrated to exhibit superior predictive capacity for IR risk compared to the TyG index alone, representing a practical, reliable, and highly reproducible novel method for assessing insulin resistance.

Evidence from multiple studies has confirmed that the risk of cardiovascular events, T2DM, non-alcoholic fatty liver disease (NAFLD) increases linearly with elevated TyG-BMI index levels (14). A study conducted in a Chinese population compared the predictive value of various TyG-related indices for CKD and found that while Triglyceride Glucose-Waist Height Ratio (TyG-WHtR) index and Triglyceride Glucose-Waist Circumference (TyG-WC) index were significantly associated with CKD, TyG-BMI did not exhibit a significant association after adjusting for confounders. This suggests that the clinical utility of different TyG-derived indices may vary. Although previous studies have investigated the relationship between the TyG index and DKD, evidence regarding the association between the composite TyG-BMI index and DKD—particularly its relationship with DKD severity—remains insufficient. Existing research has predominantly focused on the TyG index alone or has been confined to specific subpopulations (e.g., T2DM patients without NAFLD). Consequently, there is a paucity of evidence from stratified analyses examining the association between TyG-BMI and DKD severity in a broad population of hospitalized T2DM patients. Furthermore, the optimal cut-off value, sensitivity, and specificity of TyG-BMI for predicting DKD have yet to be definitively established.

Based on the aforementioned background, the present study hypothesized that: (1) the TyG-BMI index is independently associated with DKD risk in patients with T2DM; and (2) the TyG-BMI index may serve as an auxiliary reference indicator for DKD risk assessment. Therefore, this study aimed to investigate the association between the TyG-BMI index and DKD through a retrospective analysis of clinical data from hospitalized T2DM patients in China, conduct subgroup analyses according to DKD clinical stages, and evaluate the discriminatory ability of TyG-BMI for DKD using ROC curve analysis, thereby providing a reference basis for early DKD risk assessment and clinical attention.

## 2 Materials and methods

### 2.1 Ethics statement

The study was approved by the Ethics Committee of the Peking University International Hospital and was conducted in accordance with the ethics standards of institutional and national research committees and the 1964 Helsinki Declaration and its later amendments or comparable ethics standards. The study was a retrospective analysis; therefore, the requirement for written informed consent was waived.

### 2.2 Research subjects

This single-center, retrospective study enrolled patients diagnosed with T2DM who were hospitalized in Peking University International Hospital between March 2015 and August 2021.

The inclusion criteria were as follows: (1) Age ≥ 18years; (2) All participants met the 1999 World Health Organization (WHO) diagnostic criteria for T2DM (15); (3) Normal coagulation function and normal function of major organs including the heart and lungs, defined as: liver function (ALT, AST ≤ 2 × upper limit of normal), coagulation function (prothrombin time, activated partial thromboplastin time within normal range), and no history of severe cardiopulmonary disease; (4) Complete clinical data available.

The exclusion criteria were as follows: (1) Diagnosis of type 1 diabetes or other specific types of diabetes; (2) Presence of acute diabetic complications such as diabetic ketoacidosis or hyperosmolar hyperglycemic state; (3) Known primary renal parenchymal disease (e.g., glomerulonephritis, interstitial nephritis); (4) Recent urinary tract infection or current use of medications known to affect renal function (e.g., non-steroidal anti-inflammatory drugs, aminoglycoside antibiotics); (5) Concurrent severe anemia or significant blood loss; (6) Pregnancy or lactation.

### 2.3 Methods

#### 2.3.1 Clinical conditions

The following data were collected for all study participants: sex, age, duration of DM, height, weight, systolic blood pressure (SBP), diastolic blood pressure (DBP), waist circumference (WC), and hip circumference (HC). BMI = Weight (kg)/Height (m^2^). Wasit-to-hip ratio (WHR) = WC (cm)/HC (cm).

In addition, the following confounding factors were collected: smoking history, alcohol consumption, history of hypertension (HTN), history of coronary artery disease (CAD), use of glucose-lowering medications (SGLT-2 inhibitors, GLP-1 receptor agonists, etc.), antihypertensive agents (ACEI/ARB, etc.), and lipid-lowering therapies (statins).

#### 2.3.2 Laboratory biochemical indices

Laboratory biochemical indices included FPG, 2-hour postprandial plasma glucose (2hPPG), glycosylated hemoglobin (HbA1c), total cholesterol (TC), TG, low-density lipoprotein cholesterol (LDL-C), high-density lipoprotein cholesterol (HDL-C), serum uric acid (sUA), serum creatinine (sCr), estimated glomerular filtration rate (eGFR), alanine aminotransferase (ALT), aspartate aminotransferase (AST), glutamyltransferase (GGT), urinary albumin/creatinine ratio (UACR), fasting insulin (FINS), 2-hour postprandial insulin (2hINS), fasting C-Peptide (FCP), and 2-hour C-Peptide (2hCP) were detected in the clinical laboratory. The TyG-BMI index was calculated using the formula: ln [TG (mg/dL) × FPG (mg/dL)/2] × BMI (kg/m^2^).

FCP and 2hCP were included in the analysis based on the following considerations: (1) Reflection of endogenous insulin secretion function: In patients with T2DM, particularly those receiving exogenous insulin therapy, C-peptide more accurately reflects endogenous insulin secretion than insulin itself, as it is not subject to interference from exogenous insulin; (2) Pathophysiological association with IR and DKD: Previous studies (16) have demonstrated that FCP levels correlate with the degree of IR, and elevated C-peptide levels may participate in the pathogenesis and progression of DKD through mechanisms such as promoting inflammatory responses and mesangial cell proliferation. Therefore, FCP was incorporated into the analysis as a complementary indicator reflecting both pancreatic β-cell function and IR status.

#### 2.3.3 Diagnostic criteria for DKD

DKD is characterized by persistently elevated urinary albumin excretion and/or a progressive decline in kidney function. According to Clinical guideline for the prevention and treatment of diabetic kidney disease in China (2021 edition) and internationally recognized standards (17), the diagnosis is established by the presence of either a UACR ≥ 30 mg/g or an eGFR < 60 mL/min/1.73 m^2^, persisting for more than 3 months, after the exclusion of other primary causes of kidney disease.

### 2.4 Statistical analysis

All statistical analyses were performed using SPSS software (version 31.0). Continuous variables with a normal distribution and homogeneity of variance are presented as the mean ± standard deviation (x¯ ± s), while those with a skewed distribution are presented as median (interquartile range) [M (Q1, Q3)]. Categorical data are expressed as frequencies (percentages) [*n* (%)]. Intergroup comparisons for normally distributed data were performed using the Student's *t*-test. The Mann–Whitney *U* test was employed for comparisons between two groups of non-normally distributed data, and the Chi-square test was used for comparing proportions. Multiple group comparisons were performed using the least significant difference (LSD) test. Binary logistic regression analysis was conducted to identify factors influencing DKD. Variable selection followed the following strategy: (1) potential risk factors known to be associated with DKD were included in the candidate variable set based on clinical knowledge and previous literature reports; (2) variables with *P* < 0.10 in intergroup comparisons from univariate analysis were considered candidate variables; (3) to avoid overfitting, the ratio of the number of variables entered into the multivariate model to the number of events (number of DKD cases) was ensured to be at least 1:10. In this study, the number of DKD cases was 607, which could support the inclusion of up to 60 variables, satisfying model stability requirements. Variables with *P* < 0.05 in univariate analysis were entered into the multivariate logistic regression model. The stepwise forward selection method (likelihood ratio) was employed to identify significant variables, and odds ratios (OR) with their 95% confidence intervals (CI) were calculated. Confounding factors adjusted in the multivariate model included age, sex, diabetes duration, medical history (HTN and CAD), and medication history (SGLT-2 inhibitors, GLP-1 receptor agonists, ACEI/ARBs, and Statins). Prior to multivariate logistic regression analysis, multicollinearity among candidate variables was assessed using the variance inflation factor (VIF). VIF > 10 or tolerance < 0.1 indicated the presence of severe multicollinearity, necessitating consideration of variable combination or elimination. The Hosmer–Lemeshow goodness-of-fit test was employed to evaluate the calibration ability of the logistic regression model, with *P* > 0.05 indicating good model fit. Nagelkerke's R^2^ was used to assess the explanatory power of the model. Receiver operating characteristic (ROC) curve analysis was performed to evaluate the diagnostic efficacy of the TyG-BMI index for DKD, calculating the area under the curve (AUC), optimal cut-off value, sensitivity, specificity, and Youden index. All statistical tests were two-tailed, and *P* < 0.05 was considered statistically significant.

## 3 Results

### 3.1 Characteristics of study population

Herein, a total of 1,966 participants were enrolled in the study, comprising 1,237 males (62.92%) and 729 females (37.08%). Based on the DKD diagnostic criteria, participants were divided into the non-DKD group (*n* = 1,359) and the DKD group (*n* = 607). Compared to the Non-DKD group, patients in the DKD group exhibited significantly higher age, DM duration, SBP, DBP, WC, WHR, BMI, sCr, UACR, FPG, 2hPPG, HbA1c, FCP, TG, and the TyG-BMI index (*P* < 0.05). Conversely, HDL-C, e-GFR, and ALT were significantly lower in the DKD group (*P* < 0.05). Regarding comorbidities, the prevalence of CAD and HTN, as well as the usage rates of ACEI/ARBs, statins, SGLT-2 inhibitors, and GLP-1 receptor agonists, were significantly higher in the DKD group than in the non-DKD group (all *P* < 0.05) (Table 1).

**Table 1:** Comparison of general conditions and biochemical indexes between the two groups.

Variable	Non-DKD (*n* = 1,359)	DKD (*n* = 607)	t(X^2^)	*p*
Sex (%)			5.846	0.016
Male	879 (43.18)	358 (18.21)		
Female	480 (24.42)	249 (12.67)		
Age (year)	54.25 ± 13.26	57.50 ± 14.00	−4.94	0.000
DM Duration (year)	8.23 ± 7.29	11.00 ± 8.49	−7.37	0.000
Smoking	648 (47.68)	307 (50.58)	1.407	0.236
Alcohol consumption	363 (26.71)	168 (27.68)	0.199	0.656
CAD	226 (16.63)	154 (25.37)	20.559	0.000
HTN	494 (36.35)	338 (55.68)	64.250	0.000
SGLT-2 inhibitors Use	149 (10.96)	86 (14.17)	4.093	0.043
GLP-1 receptor agonists Use	124 (9.12)	74 (12.19)	4.357	0.037
ACEI/ARBs Use	399 (29.36)	247 (40.69)	24.423	0.000
Statins Use	298 (21.93)	171 (28.17)	9.004	0.003
SBP (mmHg)	130.06 ± 16.37	137.04 ± 18.07	−8.45	0.000
DBP (mmHg)	78.18 ± 10.45	78.89 ± 11.78	−3.22	0.000
WL (cm)	94.37 ± 9.63	96.76 ± 10.65	−4.91	0.000
WHR	0.94 ± 0.06	96.76 ± 10.65	−2.72	0.007
BMI (kg/m^2^)	25.69 ± 3.51	26.38 ± 4.11	−3.83	0.000
UACR (mg/g)	9.04 ± 6.61	284.47 ± 672.50	−15.10	0.000
sCr (umol/L)	66.09 ± 16.79	78.67 ± 41.34	−9.59	0.000
eGFR	100.36 ± 16.03	90.25 ± 25.25	10.70	0.000
FPG (mmol/L)	8.88 ± 3.38	9.46 ± 3.84	−3.40	0.000
2hPPG (mmol/L)	12.54 ± 4.43	13.23 ± 4.33	−3.19	0.001
HbA1C (%)	8.62 ± 1.86	8.92 ± 1.97	−3.23	0.001
ALT (U/L)	24.94 ± 14.78	23.24 ± 14.45	2.38	0.017
AST (U/L)	21.71 ± 10.01	21.67 ± 10.69	0.09	0.929
GGT (mmol/L)	36.25 ± 29.85	39.48 ± 50.37	−1.77	0.077
FCP (ng/mL)	2.34 ± 1.17	2.54 ± 1.45	−3.23	0.001
2hCP (ng/mL)	5.69 ± 3.51	5.39 ± 3.22	1.83	0.068
FINS (uU/mL)	10.69 (6.53–15.85)	13.61 (8.02–15.85)	4.680	0.000
2hINS (uU/mL)	41.40 (22.43–54.54)	44.09 (21.66–50.49)	0.156	0.876
TC (mmol/L)	4.36 ± 0.99	4.40 ± 1.26	−0.77	0.444
TG (mmol/L)	2.06 ± 1.80	2.30 ± 1.92	−2.66	0.008
HDL (mmol/L)	1.00 ± 0.23	0.97 ± 0.26	2.32	0.020
LDL (mmol/L)	2.58 ± 0.82	2.55 ± 0.94	0.731	0.465
sUA (umol/L)	337.59 ± 85.67	363.84 ± 107.75	26.00	0.000
TyG	9.30 ± 0.73	9.46 ± 0.81	−4.21	0.000
TyG-BMI	240.28 ± 39.52	250.81 ± 45.55	−5.20	0.000

### 3.2 Subgroup analysis of general conditions and biochemical indexes based on DKD severity

To further investigate the association between the TyG-BMI index and the severity of DKD, the DKD group was further stratified into two subgroups: the microalbuminuria group (DKD-1 group, UACR 30–300 mg/g, *n* = 505) and the clinical proteinuria group (DKD-2 group, UACR ≥ 300 mg/g or eGFR < 60 mL/min/1.73 m^2^, *n* = 102). Subgroup analysis revealed that, compared to the DKD-1 group, patients in the DKD-2 group had longer DM duration, higher blood pressure, and elevated levels of FPG, HbA1c, TC, TG, HDL-C, UACR, and sUA, while exhibiting lower 2hCP and eGFR (all *P* < 0.05). Although the TyG-BMI index demonstrated a gradual increasing trend with greater DKD severity, the difference between the two subgroups did not reach statistical significance (*P* = 0.562) (Table 2).

**Table 2:** Subgroup analysis of general conditions and biochemical indexes based on DKD.

Variable	Non-DKD (*n* = 1,359)	DKD-1 (*n* = 505)	DKD-2 (*n* = 102)	F(X^2^)	*p*
Sex (%)				11.058	0.004
Male	879 (44.71)^a^	308 (15.67)^ab^	50 (2.54)^b^		
Female	480 (24.42)^a^	197 (10.02)^ab^	52 (2.64)^b^		
Age (year)	54.25 ± 13.26^a^	57.13 ± 14.36^b^	59.34 ± 11.93^bc^	13.349	0.000
DM duration (year)	8.23 ± 7.29^a^	10.22 ± 8.19^b^	14.83 ± 8.98^c^	43.045	0.000
Smoking	648 (47.68)^a^	262 (51.88)^a^	45 (44.12)^a^	3.455	0.178
Alcohol consumption	363 (26.71)^a^	145 (28.71)^a^	23 (22.55)^a^	1.834	0.400
GAD	226 (16.63)^a^	130 (25.74)^b^	24 (23.53)^ab^	20.826	0.000
HTN	494 (36.35)^a^	270 (53.47)^b^	68 (66.67)^c^	70.309	0.000
SGLT-2 inhibitors Use	149 (10.96)^a^	79 (15.64)^b^	7 (6.86)^ab^	10.310	0.006
GLP-1 receptor agonists Use	124 (9.12)^a^	69 (13.66)^b^	5 (4.90)^a^	11.549	0.003
ACEI/ARBs Use	399 (29.36)^a^	200 (39.60)^b^	47 (46.08)^b^	26.036	0.000
Statins Use	298 (21.93)^a^	141 (27.92)^b^	30 (29.41)^ab^	9.108	0.011
SBP (mmHg)	130.06 ± 16.37^a^	135.34 ± 17.72^b^	145.40 ± 17.54^c^	51.483	0.000
DBP (mmHg)	78.18 ± 10.45^a^	79.27 ± 11.56^ab^	82.99 ± 12.40^c^	10.199	0.000
WL (cm)	94.37 ± 9.63^a^	96.73 ± 10.87^b^	96.90 ± 9.49^bc^	12.079	0.000
BMI (kg/m^2^)	25.69 ± 3.51^a^	26.40 ± 4.11^b^	26.27 ± 4.12^ab^	7.388	0.001
WHR	0.94 ± 0.06^a^	0.95 ± 0.06^b^	0.96 ± 0.08^bc^	4.965	0.007
UACR (mg/g)	9.04 ± 6.61^a^	87.54 ± 52.96^b^	1,259.50 ± 1,243.19^c^	924.297	0.000
sCr (umol/L)	66.09 ± 16.79^a^	74.74 ± 35.30^b^	98.14 ± 59.79^c^	80.796	0.000
eGFR	100.36 ± 16.03^a^	93.27 ± 23.53^b^	75.28 ± 28.13^c^	97.578	0.000
FPG (mmol/L)	8.88 ± 3.38^a^	9.30 ± 3.75^b^	10.25 ± 4.23^c^	8.882	0.000
2hPPG (mmol/L)	12.54 ± 4.43^a^	13.17 ± 4.11^b^	13.53 ± 5.30^bc^	5.378	0.005
HbA1C (%)	8.62 ± 1.86^a^	8.84 ± 1.94^b^	9.33 ± 2.09^c^	8.051	0.000
ALT (U/L)	24.94 ± 14.78^a^	24.23 ± 15.02^ab^	18.30 ± 9.88^c^	9.819	0.000
AST (U/L)	21.71 ± 10.01^a^	22.37 ± 11.22^ab^	18.19 ± 6.56^c^	7.142	0.001
GGT (mmol/L)	36.25 ± 29.85^a^	41.61 ± 54.51^b^	28.93 ± 16.16^ac^	6.474	0.002
FCP (ng/mL)	2.34 ± 1.17^a^	2.53 ± 1.38^b^	2.59 ± 1.76^ab^	5.328	0.005
2hCP (ng/mL)	5.69 ± 3.51^a^	5.59 ± 3.23^ab^	4.40 ± 2.97^c^	6.791	0.001
FINS (uU/mL)	14.92 ± 40.82^a^	15.70 ± 19.11^ab^	16.09 ± 26.10^abc^	0.123	0.884
2hINS (uU/mL)	48.81 ± 60.40^a^	48.35 ± 39.12^ab^	39.23 ± 45.54^abc^	1.445	0.236
TC (mmol/L)	4.36 ± 0.99^a^	4.34 ± 1.19^ab^	4.68 ± 1.53^c^	4.595	0.010
TG (mmol/L)	2.06 ± 1.80^a^	2.28 ± 1.90^bc^	2.43 ± 2.03^c^	3.841	0.022
HDL (mmol/L)	1.00 ± 0.23^a^	0.96 ± 0.24^b^	1.04 ± 0.34^ac^	6.816	0.001
LDL (mmol/L)	2.76 ± 6.74^a^	2.54 ± 0.91^ab^	2.61 ± 1.05^abc^	0.305	0.738
sUA (umol/L)	337.59 ± 85.67^a^	360.40 ± 107.77^b^	380.90 ± 106.54^c^	18.799	0.000
TyG	9.30 ± 0.73	9.43 ± 0.81	9.60 ± 0.83	11.669	0.000
TyG-BMI	240.28 ± 39.52^a^	250.33 ± 44.97^b^	253.19 ± 48.50^bc^	13.726	0.000

“a, b, c” in Table 2 indicating the results of post-hoc pairwise comparisons among the three groups. Values in the same row sharing the same superscript letter are not significantly different, whereas those with different superscript letters are significantly different (*p* < 0.05).

### 3.3 Univariate and multivariate logistic regression analysis of factors associated with DKD

Univariate logistic regression analysis revealed that sex, age, duration of DM, SBP, DBP, WHR, FBG, FCP, HbA1c, and the TyG-BMI index were significantly associated with DKD (all *P* < 0.05). Subsequently, variables with *P* < 0.05 in univariate analysis, along with clinically important confounders (smoking, alcohol consumption, HTN, CAD, medication history, sUA etc.), were entered into the multivariate logistic regression model using the stepwise forward selection method (likelihood ratio). Multicollinearity diagnostics demonstrated that the variance inflation factor (VIF) for all continuous variables was less than 5 (range: 1.12–3.48), indicating the absence of severe multicollinearity. To further assess the degree of collinearity between TyG-BMI and BMI, the Spearman correlation coefficient was calculated. The results showed a strong correlation between TyG-BMI and BMI (*ρ* = 0.82, *P* < 0.001). Nevertheless, the VIF values were all below 5, indicating that collinearity did not compromise model stability. The Hosmer–Lemeshow goodness-of-fit test for the multivariate binary logistic regression model yielded a *P*-value of 0.318, indicating good model calibration. Nagelkerke's R^2^ was 0.184, suggesting that the model explained 18.4% of the variance in DKD. After adjusting for all the above-mentioned confounders, multivariate logistic regression analysis demonstrated that the TyG-BMI index remained independently associated with DKD (OR = 1.008, 95% CI: 1.005–1.010, *P* = 0.000). To facilitate clinical interpretation, we calculated the OR per 10-unit increase in TyG-BMI, which showed that each 10-unit increase in TyG-BMI was associated with an 8.5% increase in DKD risk (OR = 1.085, 95% CI: 1.059–1.112, *P* = 0.000) (Table 3).

**Table 3:** Binary logistic regression analysis of the factors influencing DKD.

Variables	Model 1		Model 2		Model 3	
	OR (95% CI)	*P*	OR (95% CI)	*P*	OR (95% CI)	*P*
TyG-BMI (per 1-unit increase)	1.008 (1.006, 1.011)	0.000	1.007 (1.004, 1.009)	0.000	1.008 (1.005, 1.010)	0.000
TyG-BMI (per 10-unit increase)	1.085 (1.059, 1.112)	0.000	1.068 (1.041, 1.095)	0.000	1.081 (1.054, 1.108)	0.000

**Model:** ^1^ Adjusted for sex, age, and DM duration.

**Model:** ^2^ Adjusted for sex, age, DM duration, and Medical history (HTN and CAD).

**Model:** ^3^ Adjusted for sex, age, DM duration, Medical history (HTN and CAD), and Medication history (SGLT-2 inhibitors, GLP-1 receptor agonists, ACEI/ARBs, and Statins).

### 3.4 Logistic regression analysis of the relationship between quartiles of the TyG-BMI index and the occurrence of DKD

The TyG-BMI index was categorized into quartiles: Q1 (≤217.77), Q2 (217.77–244.14), Q3 (244.14–263.06), and Q4 (≥263.06). Using the lowest quartile (Q1) as the reference, logistic regression analysis was performed to evaluate the association between different TyG-BMI levels and DKD.

#### 3.4.1 The crude model (Model 1) was not adjusted for any covariates

The risk of DKD gradually increased with higher TyG-BMI quartiles. Compared to the Q1 group, the OR for DKD were 1.30 (95% CI: 0.98–1.73, *P* = 0.065) in the Q2 group, 1.43 (95% CI: 1.08–1.89, *P* = 0.012) in the Q3 group, and 2.01 (95% CI: 1.52–2.64, *P* < 0.001) in the Q4 group.

#### 3.4.2 Model 2 was adjusted for sex and age

The association between TyG-BMI index and DKD was further strengthened. Compared to the Q1 group, the OR for DKD were 1.37 (95% CI: 1.04–1.83, *P* = 0.028) in the Q2 group, 1.61 (95% CI: 1.21–2.14, *P* = 0.001) in the Q3 group, and 2.44 (95% CI: 1.84–3.25, *P* < 0.001) in the Q4 group.

#### 3.4.3 Model 3 was adjusted for sex, age, DM duration, SBP, and HbA1c

The TyG-BMI index remained significantly associated with DKD. Compared to the Q1 group, the OR for DKD were 1.35 (95% CI: 1.01–1.81, *P* = 0.042) in the Q2 group, 1.53 (95% CI: 1.14–2.04, *P* = 0.005) in the Q3 group, and 2.09 (95% CI: 1.55–2.81, *P* < 0.001) in the Q4 group (Table 4).

**Table 4:** Logistic regression analysis of the relationship between quartiles of the TyG-BMI index and the occurrence of DKD.

Stratified by Tyg-BMI quartiles	Model 1		Model 2		Model 3	
	Exp (95% CI)	*P*	OR (95% CI)	*P*	OR (95% CI)	*P*
Q1 (TyG-BMI<217.77)	1		1		1	
Q2 (217.77≤TyG-BMI<244.14)	1.303 (0.983, 1.726)	0.065	1.376 (1.035, 1.828)	0.028	1.353 (1.011, 1.812)	0.042
Q3 (244.14≤TyG-BMI<263.06)	1.428 (1.080, 1.888)	0.012	1.610 (1.211, 2.140)	0.001	1.526 (1.139, 2.044)	0.005
Q4 (TyG-BMI≥263.06)	2.005 (1.522, 2.641)	0.000	2.444 (1.836, 3.253)	0.000	2.088 (1.554, 2.805)	0.000

**Model:** ^1^ The crude model (Model 1) was not adjusted for any potential confounders.

**Model:** ^2^ Adjusted for sex, age.

**Model:** ^3^ Adjusted for sex, age, DM duration, SBP, and HbA1c.

#### 3.4.4 Trend test

To evaluate the presence of a dose-response relationship between TyG-BMI index and DKD, the TyG-BMI quartiles were included as an ordinal categorical variable (coded as Q1 = 1, Q2 = 2, Q3 = 3, Q4 = 4) in the linear trend test. The results demonstrated a significant linear trend across all models: *P* for trend < 0.001 in the crude model (Model 1), *P* for trend < 0.001 in the age- and sex-adjusted model (Model 2), and *P* for trend < 0.001 in the fully adjusted model (Model 3). These findings further support the existence of a significant dose-response trend between TyG-BMI levels and DKD risk.

### 3.5 Discriminative ability of TyG-BMI index for DKD

To assess the discriminative ability of the models for DKD, ROC curves were constructed based on the results of multivariate logistic regression analysis (Table 3). The predicted probability of the combined model (including demographic characteristics: sex and age; disease characteristics: diabetes duration, HTN, and CAD; anthropometric measurements: SBP and WC; laboratory indices: FBG, TG, HbA1c, eGFR, sUA; and the TyG-BMI index) were used as the test variables, with DKD status (presence = 1, absence = 0) as the classification variable. The combined model achieved an AUC of 0.709 (95% CI: 0.683–0.734), with a sensitivity of 58.4% and specificity of 73.4%. The optimal predicted probability cut-off for the combined model was 0.330, corresponding to a raw TyG-BMI index value of 315.962.

For comparison, we also evaluated the predictive performance of TyG-BMI alone and BMI alone. The AUC for TyG-BMI alone was 0.574 (95% CI: 0.547–0.601), with an optimal cut-off of 239.131, sensitivity of 68.7%, and specificity of 45.1%. The AUC for BMI alone was 0.552 (95% CI: 0.524–0.580), with an optimal cut-off of 25.315, sensitivity of 64.9%, and specificity of 45.4%. The DeLong test demonstrated that the AUC of the combined model was significantly superior to that of TyG-BMI alone and BMI alone (both *P* < 0.001), indicating that the addition of other clinical variables significantly improved the discriminative ability of the model (Table 5; Figure 1).

**Table 5:** The ROC curve for DKD (AUC, 95% CI, optimal cut-off value, sensitivity, specificity, and Youden index).

ROC curve	AUC	95% CI	Optimal cut-off value	Sensitivity (%)	Specificity (%)	Youden index
Combined model	0.709	(0.683, 0.734)	0.330	0.584	0.734	0.319
BMI	0.552	(0.524, 0.580)	25.315	0.649	0.454	0.103
TyG-BMI	0.574	(0.547, 0.601)	239.131	0.687	0.451	0.138

**Figure 1:**
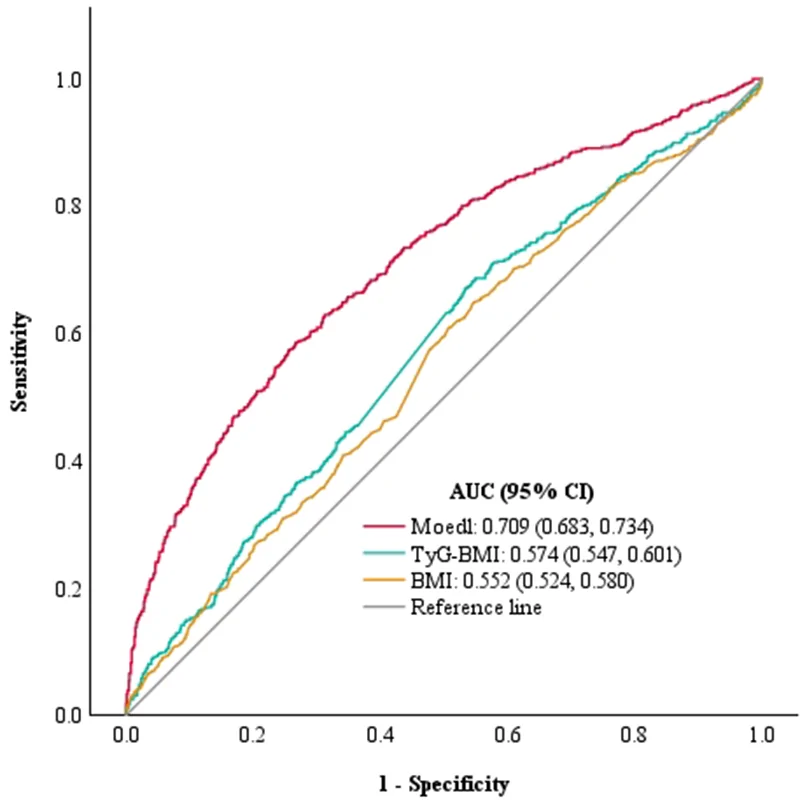
ROC curves for predicting DKD. The AUCs were 0.709 (95% CI: 0.683–0.734) for the combined model, 0.574 (95% CI: 0.547–0.601) for TyG-BMI alone, and 0.552 (95% CI: 0.524–0.580) for BMI alone. The combined model showed significantly better discriminative ability than TyG-BMI or BMI alone (DeLong test, both *P* < 0.001).

## 4 Discussion

The present study identified a significant independent association between the TyG-BMI index and DKD among hospitalized patients with T2DM. After adjusting for confounders including age, sex, diabetes duration, comorbidities (HTN and CAD), and medication history (SGLT-2 inhibitors, GLP-1 receptor agonists, ACEI/ARBs, statins), TyG-BMI remained independently associated with DKD (OR = 1.008, 95% CI: 1.005–1.010, *P* = 0.000). When TyG-BMI was stratified by quartiles, the risk of DKD exhibited a graded increase with elevating TyG-BMI levels and the trend test indicated a significant dose—response trend (*P* for trend < 0.001). Subgroup analysis showed that TyG-BMI increased with DKD severity. However, the difference between the microalbuminuria and clinical proteinuria groups did not reach statistical significance, suggesting that the relationship between TyG-BMI and DKD severity may be non-linear or modified by disease stage. ROC curve analysis showed that the combined model achieved an AUC of 0.709 (95% CI: 0.683–0.734).

IR participates in the pathogenesis and progression of DKD through multiple pathways, including inflammatory responses, oxidative stress, lipotoxicity, and mitochondrial dysfunction (18). As a composite parameter integrating glucose metabolism (FPG), lipid metabolism (TG), and obesity indicators (BMI), the TyG-BMI index may be associated with DKD through the following mechanisms.

(1) Hyperglycemia-induced renal injury. Chronic hyperglycemia can lead to glomerular and tubular damage through various pathways, including the accumulation of advanced glycation end-products (AGEs), activation of the protein kinase C pathway, and stimulation of the polyol pathway. These processes ultimately result in glomerulosclerosis, mesangial expansion, and proteinuria (19). (2) Lipid metabolism disorders and lipotoxicity. Elevated triglycerides are not only a marker of IR but may also directly damage the kidneys through lipotoxicity. The accumulation of free fatty acids in glomerular and tubular cells can activate inflammatory pathways, induce endoplasmic reticulum stress, mitochondrial dysfunction, and apoptosis, thereby promoting renal fibrosis (20). (3) Obesity-related hemodynamic alterations and inflammation. Increased BMI reflects an obese state, which can elevate the risk of kidney injury through mechanisms such as glomerular hyperfiltration, activation of the renin-angiotensin-aldosterone system, and sympathetic nervous system excitation (21). Concurrently, obesity-associated adipose tissue inflammation and adipokine imbalance (e.g., elevated leptin, decreased adiponectin) also participate in the pathogenesis of DKD (22). (4) Direct nephrotoxic effects of insulin resistance. Insulin not only acts on systemic metabolism but also directly influences the function of glomerular podocytes, mesangial cells, and tubular epithelial cells. Under IR conditions, impaired insulin signaling pathways can lead to podocyte apoptosis, foot process effacement, and disruption of glomerular filtration barrier integrity, consequently resulting in proteinuria (23, 24). Studies have demonstrated that patients with T2DM exhibiting marked IR have a significantly higher risk of developing diabetic kidney injury compared to those with milder IR (25). Furthermore, another research has shown that IR can be detected even in the early stages of renal injury in patients with T2DM (8).

The TyG-BMI index represents a novel simplified indicator for assessing IR. Compared with traditional IR assessment indices such as HOMA-IR and HOMA2-IR, this new simplified metric integrates glucose metabolism, lipid metabolism, and anthropometric measurements without requiring insulin level determination, rendering it more suitable for large-scale clinical application.

Given that TyG-BMI mathematically incorporates BMI, we performed supplementary analyses to evaluate the correlation and potential collinearity between TyG-BMI and BMI. Spearman correlation analysis showed a strong correlation between TyG-BMI and BMI (*ρ* = 0.82, *P* < 0.001), which is consistent with its mathematical composition. Nevertheless, the VIF values in the multivariate model were all <5, ranging from 1.12 to 3.48, indicating that collinearity did not substantially affect model stability. More importantly, after excluding BMI from the multivariate model, TyG-BMI remained independently associated with DKD, suggesting that its predictive value is not solely driven by BMI. These findings further support the independence of TyG-BMI as an IR assessment indicator in DKD risk prediction.

In recent years, numerous studies have investigated the relationship between TyG and its derived indices with renal injury, yet the findings have exhibited certain discrepancies, suggesting that the predictive value of different indices may vary across populations. Lv et al. (10) conducted a study involving 1,054 patients with T2DM and found that the TyG index was an independent factor associated with DKD (OR = 2.34, 95% CI: 1.78–3.08). Moreover, as TyG index levels increased, the risk of DKD progressively rose, with the highest quartile group demonstrating a 5.56-fold higher risk compared to the lowest quartile group. Similarly, Ran et al. (26) investigated 1,530 patients with T2DM and reported that the TyG index was positively correlated with diabetic nephropathy and remained an independent factor associated with DN (OR = 1.623, 95% CI: 1.175–2.242), with higher TyG levels associated with earlier onset of DN. Wang et al. (12) conducted research in a Chinese population and not only confirmed that the TyG index was an independent factor associated with DKD (OR = 1.57) but also identified a threshold effect in the relationship between TyG index and DKD risk: when the TyG index exceeded 9.35, each unit increase was associated with a 1.94-fold increase in DKD risk.

Compared to the TyG index, TyG-BMI index, as a composite parameter integrating obesity indicators—should theoretically possess superior predictive value. However, findings from existing studies are not entirely consistent. Nan Huang et al. demonstrated that the TyG-BMI index is associated with kidney impairment in patients with T2DM (27). Another study reported that in patients with newly diagnosed T2DM, the risk of DKD increased with elevating TyG-BMI index levels (28). Chu et al. (29) conducted a study involving 425 patients with T2DM and demonstrated that in patients without NAFLD, TyG-BMI index predicted DKD with an AUC of 0.78 and a sensitivity of 91.3%, suggesting that TyG-BMI index may possess superior predictive value in specific subpopulations. However, Chen et al. (30) investigated the predictive value of various TyG-related indices for CKD in a Chinese community-based population and found that while TyG-WHtR and TyG-WC were significantly associated with CKD, TyG-BMI index did not exhibit a significant association after adjusting for confounding factors. This finding suggests that the clinical utility of different TyG-derived indices may vary, with TyG-BMI index potentially demonstrating greater predictive value in specific populations (such as patients with T2DM) while exhibiting limited predictive ability in the general population.

The present study observed a significant association between TyG-BMI and DKD in hospitalized patients with T2DM. Notably,in our supplementary analysis, TyG-BMI and TyG alone showed no statistically significant difference in predictive performance for DKD (AUC: 0.574 vs. 0.558, DeLong test *P* = 0.211; combined model AUC: 0.709 vs. 0.708, *P* = 0.883). TyG-BMI did not provide significant incremental predictive value over TyG. Nevertheless, we selected TyG-BMI as the primary analysis indicator for the following reasons. First, from a pathophysiological perspective, IR is the result of the interplay among glucose metabolism, lipid metabolism, and obesity. TyG-BMI integrates these three dimensions and theoretically provides a more complete reflection of IR. Second, BMI is an easily accessible clinical indicator directly linked to lifestyle interventions. Incorporating it into a composite index allows clinicians to more intuitively associate risk assessment results with weight management and comprehensive treatment strategies. Third, in the current academic context of precision medicine and cardiovascular-kidney-metabolic (CKM) syndrome management, exploring composite parameters that integrate multidimensional indicators has important academic value. Even though our single-center data did not demonstrate a statistical advantage, this study provides new evidence regarding the differential applicability of various indicators across populations. The lack of significant difference in predictive performance between TyG-BMI and TyG in this study may be related to the characteristics of the study population—hospitalized T2DM patients have longer disease duration and more severe metabolic disturbances, and glucolipid metabolic abnormalities alone may be sufficient to drive DKD risk, with the additional contribution of BMI potentially being mediated or masked by the former. This finding suggests that both TyG and TyG-BMI can serve as effective reference indicators for DKD risk assessment in hospitalized T2DM patients, and clinicians may choose flexibly based on data availability.

After stratifying the TyG-BMI index into quartiles, we observed that the risk of DKD progressively increased with elevating TyG-BMI index levels. Across three logistic regression models, the risk of DKD in the Q4 group was 2.01-fold, 2.44-fold, and 2.09-fold higher than that in the Q1 group, respectively. This study demonstrated a significant dose-response trend between the TyG-BMI index and DKD. This trend remained significant even after adjusting for confounding factors (*P* for trend < 0.001). Notably, although patients in the clinical proteinuria group exhibited more severe metabolic disturbances, the difference in TyG-BMI index between the two subgroups did not reach statistical significance (*P* = 0.562). This negative finding warrants in-depth discussion. This phenomenon may be attributed to the following factors. First, the relatively small sample size of the clinical proteinuria group (*n* = 102) may have limited statistical power. Second, as a composite indicator of IR, TyG-BMI index may be influenced by multiple confounding factors in patients with advanced DKD. Studies have demonstrated complex interactions among nutritional status, lipid metabolism disorders, and IR in patients with renal failure. Fritz et al. (31) in a prospective study involving over 100,000 participants, found that while the TyG index mediated 33% of the association between BMI and ESRD, the complex network of metabolic factors may render the relationship between single indicators and outcomes non-linear upon entering the ESRD stage. Similarly, Khor et al. (32) investigated maintenance hemodialysis patients and reported that as renal function declines—particularly in ESRD—patients frequently develop protein-energy wasting and malnutrition. These factors alter the lipid metabolism profile, potentially disrupting the linear association between the TG-based TyG-BMI and the severity of IR and kidney injury. Furthermore, in the present study, 2hCP levels were significantly lower in the clinical proteinuria group compared to the microalbuminuria group, suggesting progressive pancreatic β-cell dysfunction in patients with advanced DKD. Xie et al. (33) utilizing NHANES database, stratified patients with T2DM into different subgroups and discovered that patients in the severe insulin deficiency subgroup exhibited marked β-cell failure. However, their TyG index was not the highest. This finding suggests that under conditions of severe insulin secretion deficiency, TyG-related indicators calculated based on FPG and TG may not accurately reflect the true state of IR. Chen et al. (34) further elucidated that when β-cell function fails, FPG increases significantly. However, this elevation primarily reflects insulin secretion deficiency rather than IR. In summary, the lack of a significant linear association between TyG-BMI and DKD severity in the subgroup analysis may reflect the limitations of this indicator in advanced DKD stages, rather than negating its overall association with DKD. This finding suggests that the relationship between TyG-BMI and DKD may be non-linear or modified by disease stage.

The ROC curve analysis in the present study demonstrated that, after adjusting for demographic characteristics, disease characteristics, anthropometric measurements, and laboratory indices, the combined model achieved an AUC of 0.709 (95% CI: 0.683–0.734) for diagnosing DKD, with a sensitivity of 58.4% and specificity of 73.4%. The optimal predicted probability cut-off for the combined model was 0.330, corresponding to a raw TyG-BMI index value of 315.962. This predictive performance is comparable to findings from previous studies. Mu et al. (35) investigated newly diagnosed T2DM populations and reported that TyG-BMI predicted DKD with an AUC of 0.639, which was comparable to the predictive ability of other non-insulin-based IR indices (TyG index, TG/HDL ratio, and METS-IR), ranging from 0.639 to 0.652. It should be noted that an AUC of 0.709 indicates acceptable discriminative ability, but its predictive accuracy at the individual level remains limited. Therefore, TyG-BMI is better suited as an auxiliary reference indicator to be used in combination with other clinical factors, rather than as an independent DKD screening tool.

This study is the first to systematically investigate the stratified association between TyG-BMI and DKD severity in hospitalized Chinese patients with T2DM, and to determine the optimal predicted probability cut-off of 0.330 for the combined model, corresponding to a raw TyG-BMI index value of 315.962. Through multi-model sensitivity analyses and trend tests, we confirmed the existence of a dose-response trend, thereby providing a reference basis for early DKD risk assessment and clinical attention. These insights are particularly relevant for assessing DKD risk in individuals with prediabetes and undiagnosed diabetes based on TG, FPG, and BMI levels. For high-risk populations, early interventions targeting dyslipidemia, hyperglycemia, and body weight should be implemented to reduce the risk of DKD and its progression to end-stage renal disease.

However, the present study has several limitations that should be acknowledged. First, this study only enrolled hospitalized patients with T2DM, which may introduce selection bias into the study population. Hospitalized patients typically present with more severe disease, more comorbidities, and longer disease duration, which may have led to an overestimation of the association between TyG-BMI and DKD. Therefore, caution is warranted when extrapolating the results to community-based patients with milder disease. Whether the association between TyG-BMI and DKD observed in this study also exists in community-based T2DM patients warrants further validation through future community-based studies. Second, although the diagnosis of DKD was based on internationally recognized UACR and eGFR criteria, and patients with known primary renal diseases, recent urinary tract infections, or use of nephrotoxic medications were excluded, the lack of pathological confirmation via renal biopsy may still introduce diagnostic bias, particularly in patients with concomitant HTN or primary kidney disease. Although all enrolled patients underwent routine urinalysis and renal ultrasonography to exclude known renal pathologies, the retrospective design precluded systematic screening for NDRD in every patient. Third, regarding confounding factors, in this revision we retrieved data on medication history (glucose-lowering, antihypertensive, and lipid-lowering agents), lifestyle information (smoking, alcohol consumption), and comorbidities (HTN duration and control status, CAD, et al.) for all 1,966 patients, and incorporated them into the multivariate model for adjustment. The results still showed that TyG-BMI was independently associated with DKD. However, other unmeasured residual confounding factors (such as dietary structure, physical activity level, etc.) that may affect the results cannot be completely excluded in a retrospective study. Fourth, this was a single-center cross-sectional study conducted in hospitalized patients, which can only reveal the correlation between TyG-BMI and DKD but cannot establish causality between the two or support predictive inferences. Furthermore, the study participants were recruited from a single hospital in Beijing, China, which may introduce regional and population specificity; therefore, caution is warranted when extrapolating the study conclusions to other regions or populations. Future multicenter prospective cohort studies are needed to validate the causal pathways.

## 5 Conclusion

Despite the aforementioned limitations, this study possesses certain clinical significance. The TyG-BMI index is composed of three simple, economical, and highly accessible indicators—TG, FBG, and BMI—thereby constructing a low-cost assessment model through the integration of routine biochemical parameters and body weight measurements. The present study identified TyG-BMI as an independent factor associated with DKD in patients with T2DM, demonstrating a dose-response trend with DKD risk. TyG-BMI levels were significantly elevated in patients with DKD, and higher TyG-BMI levels were associated with increased DKD risk. These findings suggest that TyG-BMI may serve as an auxiliary reference indicator for DKD risk assessment, facilitating the identification of high-risk individuals and prompting clinical attention. However, it must be emphasized that the cross-sectional design of this study does not support predictive or causal inferences, and TyG-BMI should not be regarded as an independent DKD screening tool. These findings provide preliminary evidence supporting the potential application of TyG-BMI in risk assessment for diabetic microvascular complications, but its clinical utility requires further validation through multicenter prospective studies.

## Data Availability

The raw data supporting the conclusions of this article will be made available by the authors, without undue reservation.

## References

[1] Sun H Saeedi P Karuranga S Pinkepank M Ogurtsova K Duncan BB. IDF Diabetes Atlas: global, regional and country-level diabetes prevalence estimates for 2021 and projections for 2045. Diabetes Res Clin Pract. (2022) 183:109119. 10.1016/j.diabres.2021.109119 34879977 PMC11057359

[2] Wang L Peng W Zhao Z Zhang M Shi Z Song Z. Prevalence and treatment of diabetes in China, 2013–2018. JAMA. (2021) 326:2498–506. 10.1001/jama.2021.22208 34962526 PMC8715349

[3] Guo L. 2021 annual review of major progress in diabetes. Chin J Diabetes Mellitus. (2022) 14:1–8.

[4] American Diabetes Association Professional Practice Committee. 11. Chronic kidney disease and risk management: standards of care in diabetes-2026. Diabetes Care. (2026) 49(Suppl 1):S246–60. 10.2337/dc26-S011 41358881 PMC12690176

[5] Kidney Disease: Improving Global Outcomes (KDIGO) CKD Work Group. KDIGO 2024 clinical practice guideline for the evaluation and management of chronic kidney disease. Kidney Int. (2024) 105:S117–314. 10.1016/j.kint.2023.10.018 38490803

[6] Fox CS Matsushita K Woodward M Bilo HJ Chalmers J Heerspink HJ. Associations of kidney disease measures with mortality and end-stage renal disease in individuals with and without diabetes: a meta-analysis. Lancet. (2012) 380:1662–73. 23013602 10.1016/S0140-6736(12)61350-6 PMC3771350

[7] Gupta S Dominguez M Golestaneh L. Diabetic kidney disease: an update. Med Clin North Am. (2023) 107:689–705. 10.1016/j.mcna.2023.03.004 37258007

[8] Svensson M Eriksson JW. Insulin resistance in diabetic nephropathy–cause or consequence? Diabetes Metab Res Rev. (2006) 22:401–10. 10.1002/dmrr.648 16703644

[9] Guerrero-Romero F Simental-Mendía LE González-Ortiz M Martínez-Abundis E Ramos-Zavala MG Hernández-González SO. The product of triglycerides and glucose, a simple measure of insulin sensitivity. Comparison with the euglycemic-hyperinsulinemic clamp. J Clin Endocrinol Metab. (2010) 95:3347–51. 10.1210/jc.2010-0288 20484475

[10] Lv L Zhou Y Chen X Gong L Wu J Luo W. Relationship between the TyG index and diabetic kidney disease in patients with type-2 diabetes mellitus. Diabetes Metab Syndr Obes. (2021) 14:3299–306. 10.2147/DMSO.S318255 34305401 PMC8296712

[11] Zhang F Han Y Mao Y Li W. Associations between non-insulin-based insulin resistance indices and diabetic nephropathy in patients with diabetes mellitus in US adults: a cross-sectional study of NHANES 1999−2018. Front Endocrinol (Lausanne). (2024) 15:1458521. 10.3389/fendo.2024.1458521 39720248 PMC11666371

[12] Wang H Chen G Sun D Ma Y. The threshold effect of triglyceride glucose index on diabetic kidney disease risk in patients with type 2 diabetes: unveiling a non-linear association. Front Endocrinol (Lausanne). (2024) 15:1411486. 10.3389/fendo.2024.1411486 38938513 PMC11208310

[13] Sharifi M Sedgi FM Soleimani E Heshmatipoor H Shirani M Askari G. Investigating the association between triglyceride-glucose index and risk of chronic kidney disease: a systematic review and meta-analysis. Clin Nutr ESPEN. (2025) 69:154–66. 10.1016/j.clnesp.2025.06.028 40562097

[14] Song K Xu Y Wu S Zhang X Wang Y Pan S. Research status of triglyceride glucose-body mass index (TyG-BMI index). Front Cardiovasc Med. (2025) 12:1597112. 10.3389/fcvm.2025.1597112 40756600 PMC12315701

[15] Alberti KG Zimmet PZ. Definition, diagnosis and classification of diabetes mellitus and its complications. Part 1: diagnosis and classification of diabetes mellitus provisional report of a WHO consultation. Diabetic Med. (1998) 15:539–53.10.1002/(SICI)1096-9136(199807)15:7<539::AID-DIA668>3.0.CO;2-S 9686693 10.1002/(SICI)1096-9136(199807)15:7<539::AID-DIA668>3.0.CO;2-S

[16] Chen J Huang Y Liu C Chi J Wang Y Xu L. The role of C-peptide in diabetes and its complications: an updated review. Front Endocrinol (Lausanne). (2023) 14:1256093. 10.3389/fendo.2023.1256093 37745697 PMC10512826

[17] KDOQI. KDOQI clinical practice guidelines and clinical practice recommendations for diabetes and chronic kidney disease. Am J Kidney Dis. (2007) 49:S12–154. 10.1053/j.ajkd.2006.12.005 17276798

[18] Chen X Shi C Wang Y Yu H Zhang Y Zhang J. The mechanisms of glycolipid metabolism disorder on vascular injury in type 2 diabetes. Front Physiol. (2022) 13:952445. 10.3389/fphys.2022.952445 36117707 PMC9473659

[19] Zhang H Wang K Zhao H Qin B Cai X Wu M. Diabetic kidney disease: from pathogenesis to multimodal therapy-current evidence and future directions. Front Med (Lausanne). (2025) 12:1631053. 10.3389/fmed.2025.1631053 40861214 PMC12370670

[20] Yu W Haoyu Y Ling Z Xing H Pengfei X Anzhu W. Targeting lipid metabolic reprogramming to alleviate diabetic kidney disease: molecular insights and therapeutic strategies. Front Immunol. (2025) 16:1549484. 10.3389/fimmu.2025.1549484 40352935 PMC12061959

[21] Sebastian SA Padda I Johal G. Cardiovascular-kidney-metabolic (CKM) syndrome: a state-of-the-art review. Curr Probl Cardiol. (2024) 49:102344. 10.1016/j.cpcardiol.2023.102344 38103820

[22] Gerdanovics CA Suciu ȘM Orășan OH Para I Turi VR Milaciu MV. Adipokines and vascular modulators in CKM: emerging biomarkers as diagnostic and therapeutic targets. Int J Mol Sci. (2025) 26:11083. 10.3390/ijms262211083 41303567 PMC12652559

[23] Artunc F Schleicher E Weigert C Fritsche A Stefan N Häring HU. The impact of insulin resistance on the kidney and vasculature. Nat Rev Nephrol. (2016) 12:721–37. 10.1038/nrneph.2016.145 27748389

[24] Pina AF Borges DO Meneses MJ Branco P Birne R Vilasi A. Insulin: trigger and target of renal functions. Front Cell Dev Biol. (2020) 8:519. 10.3389/fcell.2020.00519 32850773 PMC7403206

[25] Ahlqvist E Storm P Käräjämäki A Martinell M Dorkhan M Carlsson A. Novel subgroups of adult-onset diabetes and their association with outcomes: a data-driven cluster analysis of six variables. Lancet Diabetes Endocrinol. (2018) 6:361–9. 10.1016/S2213-8587(18)30051-2 29503172

[26] Ran L Han Y Zhaohu H Hailin S. Correlation between triglyceride-glucose index and microvascular complications in patients with early-onset of type 2 diabetes mellitus. Endocrinol Diabetes Metab. (2025) 8:e70027. 10.1002/edm2.70027 39946246 PMC11824366

[27] Huang N Lu B Zhu ZZ Zhu XY Chen S Shu ZY. The association between triglyceride glucose-body mass index and kidney impairment in patients with type 2 diabetes mellitus. Diabetes Metab Syndr Obes. (2024) 17:3447–53. 10.2147/DMSO.S477836 39309307 PMC11414635

[28] Jiang Y Lai X. Association between the triglyceride glucose index, triglyceride-glucose body mass index and diabetic kidney disease in adults with newly diagnosed type 2 diabetes. Front Med (Lausanne). (2024) 11:1328601. 10.3389/fmed.2024.1328601 38721354 PMC11076669

[29] Chu Y Yao H Lu N Chang Y Wang J Xu L. Triglyceride glucose-body mass index is associated with diabetic kidney disease in type 2 diabetes mellitus patients without non-alcoholic fatty liver disease. Front Nutr. (2025) 12:1628867. 10.3389/fnut.2025.1628867 40740642 PMC12307136

[30] Chen T Liu Y Wu S Long S Feng L Lu W. Comparison of TyG and newly TyG related indicators for chronic kidney diseases estimation in a Chinese population. Diabetes Metab Syndr Obes. (2024) 17:3063–75. 10.2147/DMSO.S469260 39184550 PMC11342947

[31] Fritz J Brozek W Concin H Nagel G Kerschbaum J Lhotta K. The association of excess body weight with risk of ESKD is mediated through insulin resistance, hypertension, and hyperuricemia. J Am Soc Nephrol. (2022) 33:1377–89. 10.1681/ASN.2021091263 35500938 PMC9257805

[32] Khor B-H Sahathevan S Sualeheen A Ali MSM Narayanan SS Chinna K. Circulating fatty acid profiles are associated with protein energy wasting in maintenance hemodialysis patients: a cross-sectional study. Sci Rep. (2021) 11:1416. 10.1038/s41598-020-80812-1 33446880 PMC7809126

[33] Xie J Zhang X Shao H Jing S Shan T Shi Y. An affordable approach to classifying type 2 diabetes based on fasting plasma glucose, TyG index and BMI: a retrospective cohort study of NHANES data from 1988 to 2014. Diabetol Metab Syndr. (2022) 14:113. 10.1186/s13098-022-00883-0 35948978 PMC9364489

[34] Chen Z Wen J. Elevated triglyceride-glucose (TyG) index predicts impaired islet β-cell function: a hospital-based cross-sectional study. Front Endocrinol (Lausanne). (2022) 13:973655. 10.3389/fendo.2022.973655 36246870 PMC9563389

[35] Mu X Wu A Hu H Yang M Zhou H. Correlation between alternative insulin resistance indexes and diabetic kidney disease: a retrospective study. Endocrine. (2024) 84:136–47. 10.1007/s12020-023-03574-6 37906402

